# Investigations into the presence of nidoviruses in pythons

**DOI:** 10.1186/s12985-020-1279-5

**Published:** 2020-01-17

**Authors:** Silvia Blahak, Maria Jenckel, Dirk Höper, Martin Beer, Bernd Hoffmann, Kore Schlottau

**Affiliations:** 1 Chemical and Veterinary Investigation Office, Westerfeldstraße 1, D-32758 Detmold, Germany; 2 Institute of Diagnostic Virology, Friedrich-Loeffler-Institut, Südufer 10, D-17493 Greifswald-Insel Riems, Germany; 3 Commonwealth Scientific and Industrial Research Organisation, Canberra, ACT 2601 Australia

**Keywords:** Nidovirus, Python, Snake, Respiratory disease, Pneumonia, RT-qPCR, Reptiles, High-throughput sequencing

## Abstract

**Background:**

Pneumonia and stomatitis represent severe and often fatal diseases in different captive snakes. Apart from bacterial infections, paramyxo-, adeno-, reo- and arenaviruses cause these diseases. In 2014, new viruses emerged as the cause of pneumonia in pythons. In a few publications, nidoviruses have been reported in association with pneumonia in ball pythons and a tiger python. The viruses were found using new sequencing methods from the organ tissue of dead animals.

**Methods:**

Severe pneumonia and stomatitis resulted in a high mortality rate in a captive breeding collection of green tree pythons. Unbiased deep sequencing lead to the detection of nidoviral sequences. A developed RT-qPCR was used to confirm the metagenome results and to determine the importance of this virus. A total of 1554 different boid snakes, including animals suffering from respiratory diseases as well as healthy controls, were screened for nidoviruses. Furthermore, in addition to two full-length sequences, partial sequences were generated from different snake species.

**Results:**

The assembled full-length snake nidovirus genomes share only an overall genome sequence identity of less than 66.9% to other published snake nidoviruses and new partial sequences vary between 99.89 and 79.4%. Highest viral loads were detected in lung samples. The snake nidovirus was not only present in diseased animals, but also in snakes showing no typical clinical signs.

**Conclusions:**

Our findings further highlight the possible importance of snake nidoviruses in respiratory diseases and proof multiple circulating strains with varying disease potential. Nidovirus detection in clinical healthy individuals might represent testing during the incubation period or reconvalescence. Our investigations show new aspects of nidovirus infections in pythons. Nidoviruses should be included in routine diagnostic workup of diseased reptiles.

## Background

Snakes have become an increasingly popular exotic pet in the last decades. Especially snakes of the family Boidae, which includes the so-called boas and pythons, are kept in high numbers in captivity. These snakes are non-venomous and most of them are easy to handle. Owners appreciate their fascinating colors, the considerable size of some the species and interesting physiology. Most of these snakes are bred in captivity but new species, new morphs and fresh bloodlines for breeding stocks are imported from the wild. This offers the opportunity for an exchange of different pathogens between animals from different ecologically and geographically regions. Therefore, more and more reptile diseases have been detected and for some of them pathogens were identified. Pneumonia and stomatitis are common problems in captive snakes and can be caused by different infectious agents. Mostly, they are linked to bacteria, but often a viral disease represents the basic infection. Viruses infecting reptiles and especially snakes causing respiratory signs include paramyxoviruses, reo-, adeno- and arenaviruses. These findings are mostly based on clinical, pathological and histological observations [1–3].

In 2014, three working groups described contemporaneous using unbiased deep sequencing a new nidovirus in captive pythons suffering from pneumonia. The first report from the USA investigated material from eight deceased ball pythons (*Python regius*) with a pneumonia. By deep sequencing, they were able to obtain a complete coding genome of a novel nidovirus. As a control, organ materials of 57 snakes, mostly colubrid, with clinical signs other than respiratory diseases were tested [4]. The other study from the USA focused as well on ball pythons. There, 12 snakes suffering from pneumonia, tracheitis and esophagitis were examined and were subjected to a metagenomic workflow and partial sequences from a novel nidovirus were derived [5]. The third study described the results from a diagnostic workup of an Indian python (*Python molurus*) diagnosed with severe necrotizing pneumonia from a zoological garden in northern Germany. Again, unbiased sequencing resulted in the complete coding genome sequence of a novel nidovirus, which could not be detected in ten healthy boas [6]. After that, a working group from Switzerland detected a nidovirus in different captive breeding colonies of green tree pythons (*Morelia viridis*). Twelve deceased animals with pneumonia were investigated and this virus shared only < 85% sequence similarity to the previous published sequences [7]. Marschang and Kolesnik detected the virus not only in deceased animals, but also in swab samples from living animals. Out of the 201 examined animals, 30 were RNA positive. The animals originated from different parts of Europe and positive animals included *Python regius*, *Python molurus*, *Morelia viridis*, *Morelia spilota* and *Boa constrictor*. Pythons (27.4%) were more affected than boas (2.4%) [8]. In Italy the virus was detected in six tracheobronchial lavages from diseased ball pythons [9]. A working group in the USA fulfilled Koch’s postulates in 2018, which showed a causal relationship between the novel snake nidovirus and pneumonia in ball pythons by experimentally infection of three animals. The observed clinical signs covered oral mucosal redding, mucous secretions, open-mouth breathing and anorexia. Histologic examinations showed rhinitis, stomatitis, tracheitis, esophageitis and interstitial pneumonia. The virus was present in oral secretions as well as in feces, suggesting either transmission by aerosolization or by the fecal-oral route [10]. Besides these reports from snakes, nidoviruses were also found in other reptiles like turtles and lizards in the last years. In lizards, they have been proven in animals with and without clinical signs. In turtles, the virus was found only once in diseased animals [11, 12].

The order *nidovirales* compromises divergent virus families with notable human and animal pathogens like for example severe acute respiratory syndrome (SARS) coronavirus and porcine reproductive and respiratory syndrome virus (PRRSV) [13]. The snake nidoviruses were assigned to the family *coronaviridae* in the subfamily *torovirinae* that covers two genera: *torovirus* and *bafiniviru*s infecting mammals and fish, respectively [14–17]. The reptile nidoviruses cluster together in a monophyletic clade and a new genus for these unassigned viruses was proposed: *barnivirus* (bacilliform reptile nidovirus) [4]. Last year the ICTV changed the taxonomy of the order *nidovirales* from four to seven suborders. The subfamily *torovirina*e, based on molecular properties, is now no longer part of the *coronaviridae*, but belongs instead to a suborder called *tornidovirineae* in a new family called *tobaniviridae*. Within this family, the subfamily *Serpentovirinae* compromises in the genus *Roypretovirus* the ball python nidovirus (ICTV, 2018).

In this study, we report the detection of divergent snake nidoviruses after metagenomics analysis and their distribution in different snake species, which is not always correlating with disease signs.

## Materials and methods

### Sample collection

In 2014, several snakes in a collection of Green Tree pythons suffered from severe stomatitis and pneumonia (up to 25 animals during several weeks). Bacteriological investigations of oral swabs yielded different results in the diseased snakes. Mycological and virological investigations (paramyxovirus, arenavirus, reovirus, adenovirus) were negative. Direct microscopic evaluation of mucus showed no parasites; parasitological investigations of feces were also negative. The stomatitis was treated locally (cleansing, antibiotic ointments or fluids). For the pneumonia, a systemic treatment with antibiotics was carried out according to an antibiogram following bacteriological investigations of oral swabs. As a supportive treatment, infusions (Ringer’s solution, 10 ml/kg) and ZylexisR were applied (twice in a week interval). MetacamR was used to calm down the inflammation. Despite this therapy, most of the infected snakes died or had to be euthanized (20 out of a collection of 60 snakes).

A total of 1554 captive boid snakes were screened for snake nidoviruses. The available samples included organ tissue samples (mostly lung, in some cases liver, kidney, small intestine, brain and pancreas) from deceased animals (230) as well as oral swabs or tracheal washes from living snakes (1324). Some animals were tested at several time points.

The oral swabs and tracheal washes were sent to the institute between 2015 and 2018 from different parts of Germany (all 16 federal states) and neighboring countries. This includes oral swabs from Denmark (78), 15 samples from a collection in Italy, 20 samples from five different collections in France and 117 samples from seven collections in Austria. Some of the animals showed typical symptoms (stomatitis and / or pneumonia), others without any symptoms were investigated to obtain an overview of the infection status of the collection.

### Pathological examinations

Nine green tree snakes were sent for necropsy. Diagnostic workup in the chemical and veterinary investigation office included a gross pathology, histopathology, virology and bacteriology. Necropsy and following investigations have been carried out using standard techniques. In short, after pathological examination of the carcasses tissues were fixed in formalin and stained with Haematoxylin-Eosin. The tissues were evaluated microscopically. All snakes were investigated virologically. Suspensions of liver, lung, kidney and intestine were inoculated bacteria-free into Viper Heart Cells at 29 °C (ATCC CCL 140) and incubated for 1 week. Supernatant was transferred to a new cell culture and incubated for another week. Cells were inspected daily for the presence of cytopathic effects. PCRs for paramyxovirus [18], for reovirus [19], for reptarenavirus [20], ranavirus [21] and for adenovirus [22] were performed with organ tissues and cell culture supernatant. Bacteriological investigations from liver, kidney and lung were carried out in five snakes onto blood agar, incubated at 30 °C. Isolated bacteria were typed using MALDI TOF.

### RNA extraction for NGS and RT-qPCR

Small pieces of tissue samples were homogenized in 1 mL PBS with a 5 mm steel bead in a TissueLyserII (Qiagen, Hilden, Germany). Dry swab samples taken from the trachea of the snakes were resuspended in 2 ml cell culture medium by shaking for half an hour at room temperature. For metagenomics analysis 250 μl of homogenized lung tissue was mixed with 750 μl Trizol Reagent (life technologies, Darmstadt, Germany). Afterwards chloroform was added and RNA from the aqueous phase was precipitated with 75% ethanol. RNA was further purified with RNeasy Mini Kit (QIAGEN, Hilden, Germany) according to the manufacturer’s instructions and eluted in 50 μl RNase free water. For screening purposes nucleic acids from tissue or swab samples were extracted using the King Fisher 96 Flex (Thermo Fisher Scientific, Braunschweig, Germany) in combination with the NucleoMagVet kit (Macherey-Nagel, Düren, Germany) according to the manufacturer’s instructions and eluted in 100 μl. 

### Next-generation sequencing

Sample material from three deceased snakes was analyzed by a metagenomics workflow as described elsewhere [23, 24].

### Conventional RT-PCR

In order to find specific primers for one published ball python nidovirus (KJ541759) [4] the primer designed tool Primer-BLAST was used [25]. Three different primer pairs were tested. The RT-PCR was done with the One step RT-PCR kit (Qiagen, Hilden, Germany) and uses a forward primer (5’CAA CTC TGC ACA AAC GCG AA 3′) and a reverse primer (5’CGG CGA TCT TGA TGT TGC TG 3′) amplifying a PCR product of approximately 300 bp. The temperature profile consists of a reverse transcription step at 50 °C for 45 min, an activation step at 94 °C for 15 min, followed by 40 cycles of 94 °C for 30 s, 57 °C for 45 s and 72 °C for 45 s as well as a final extension step at 72 °C for 5 min. The PCR products were visualized by electrophoreses on an agarose gel.

### Snake nidovirus specific RT-qPCR

To confirm the results of the metagenomic workflow and to screen for further infected animals, a real-time RT-PCR for the detection of snake nidoviruses was developed. Primers and probes targeting the replicase open reading frame 1B (ORF1B) were selected based on an alignment of published sequence information (NCBI GenBank) together with the two newly generated complete coding sequences from this study. The PCR was performed with the AgPath-IDTM One-Step RT-PCR kit (Thermo Fisher Scientific, Braunschweig, Germany) and a snake nidovirus specific FAM-labelled primer-probe mix consisting of 800 nM Nido-Snake-20,528-F (5′ ACA TCT CGA GAC SAT YAT CCA 3′), 800 nM Nido-Snake-20,616-R (5′ CTG TAC TWG AAC AGA AYT CGT G 3′) and 200 nM Nido-Snake-20,579-FAM-as (5′ FAM-TTC CCA MGC YTT GTT CTS GTC GAC-BHQ1 3′) was used for broad-range nidovirus detection producing a 89 bp long fragment. For the RT-qPCR reaction, 10 μl master mix and 2.5 μl RNA were combined in a total reaction volume of 12.5 μl. The PCR was carried out using a Bio-Rad CFX 96 Real-Time Detection System (Bio-Rad, Hercules, CA, USA) and the following temperature profile: 10 min at 45 °C for reverse transcription, 10 min at 95 °C for denaturation and 45 cycles of 15 s at 95 °C, 30 s at 56 °C and 30 s at 72 °C. In addition, a HEX-labelled internal control assay was used to monitor for efficient nucleic acid extraction [26].

### Snake retrovirus specific SYBR green PCR

The metagenomics workflow provided some reads of snake nidoviruses. To see how frequent these viruses occur, a SYBR green based screening RT-qPCR was developed. The RT-qPCR reaction was prepared using a SensiFAST SYBR No-Rox Kit (Bioline, London, United Kingdom) in a volume of 10 μl including 400 nM Snake_RV_2624-F (5′ ACA GTG CCT GAC CCA TAC AC 3′), 400 nM Snake_RV_2716-R (5′ AAG ACC AAA ATG CAT CTT TCA GAT C 3′), 400 nM Snake_RV_2773-R (5′ TGT ATC TGG GTC AGT CCA TTC AA 3′) and 2 μl of extracted RNA. The reaction was performed for 10 min at 45 °C for reverse transcription, 2 min at 95 °C for activation of the polymerase, and 45 cycles of 5 s at 95 °C and 20 s at 60 °C followed by a melting curve analysis. The PCR was carried out using a Bio-Rad CFX 96 Real-Time Detection System (Bio-Rad, Hercules, CA, USA).

### Dideoxy chain termination sequencing

For phylogenetic analysis, primers were designed to generate a partial sequence of the ORF1AB RNA-dependet-RNA-polymerase gene by dideoxy chain-termination sequencing [27] from representative snake nidovirus RT-qPCR-positive samples. The amplification reaction was performed using the SuperScript III One-step RT-PCR Kit (Thermo Fisher Scientific, Waltham, Massachusetts, USA) combined with the primers Nido-Snake-19,971-F (5′ ATC GGA GTC WCA AAA TTC CGA G 3′) and Nido-Snake-21,007-R (5′ CAC GTR TAG CAY TGC TGC TG 3′). For the amplification, 800 nM of each primer in 12.5 μl total reaction volume including 2.5 μl template RNA was used. The amplified PCR fragments were separated on agarose gels, visualized by staining with ethidium bromide and subsequently excised and purified using the QIAquick Gel Extraction kit (Qiagen, Hilden, Germany). Sequencing reactions of both strands were carried out with the primers used for amplification and the BigDye Terminator v1.1 Cycle Sequencing Kit (Thermo Fisher Scientific) on a 3130 Genetic Analyzer (Thermo Fisher Scientific).

### Phylogenetic analysis

The newly generated sequences of two full-length snake nidoviruses were aligned to each other as well as to further complete coding genome sequences from different members of the subfamily *Tobaniviridae* obtained from GenBank using the method MAFFT [28] as implemented in the Geneious software (version 10.2.1). Furthermore, the 36 partial ORF1AB sequences were aligned to each other and to other full-length snake nidovirus genomes. Based on these alignments, maximum-likelihood trees (PhyML) were calculated using the HKY85 model [29] with 1000 bootstrap replicates by the Geneious software [30].

## Results

### Pathological findings

Nine green tree pythons were sent for necropsy. Four snakes were investigated pathohistologically, bacteriologically and virologically in detail (Table 1). After the first results indicated a viral aetiology, five more were examined only pathohistologically and virologically. All snakes revealed pneumonia of varying degrees. Five of the snakes also showed a diphtheroid-necrotizing stomatitis, and four suffered from renal gout (Table 1). The results of the bacteriological studies showed different germs. Paramyxo-, Reo-, Adeno- and Arenavirus were excluded by PCR and/or cell culture. Attempts to isolate viruses in cell culture were unsuccessful.

**Table 1:** Detailed pathological, histological, and bacteriological findings of four green tree pythons (*Morelia viridis*)

Sample number	Sex	Pathology	Histology	Bacteriology
BH128/14–7-10	male	purulent stomatitis, lung: Dark red parenchyma, filled with yellow exudate. Stomach: empty Small intestine: slightly filled with mushy ingesta Cecum, large intestine: empty	heart, small intestine: no pathological findings.	
			lungs: high-grade purulent-necrotizing pneumonia Ziehl-Neelsen-staining lung: acid-fast rods were not detected	lung: Bordetella hinzii (+++) additionally after 48 h incubation: Providencia rettgeri
			liver: multifocal encapsulated necrosis with low inflammatory response	liver: Acinetobacter pitii (+), Providencia rettgeri (++)
			kidney: low grade renal gout	kidney: Acinetobacter pitii (+) after 48 h, Providencia rettgeri (+)
			esophageal tonsils: High grade purulent necrotizing inflammation	inhibitor substance test: positive
BH128/14–11-14	male	oral cavity: purulent stomatitis Lung: dark red, filled with exudate Stomach: empty Small intestine: slightly filled with mushy ingesta cecum, large intestine: empty	heart, liver: no pathological findings	liver: Stenotrophomonas maltophilia (+++)
			lungs: purulent necrotizing pneumonia	lung: Stenotrophomonas maltophilia (+++)
			kidney: slight uric acid deposits	kidney: Stenotrophomonas maltophilia (+) after 48 h incubation
			small intestine, pancreas: autolysis	inhibitor substance test: positive
BH128/14–1-6	female	oral cavity: purulent deposits in the periodontal pockets and on the mucous membrane Heart: artifacts due to euthanasia Lung: Slightly filled with exudate Stomach: empty Small bowel: empty Cecum, large intestine: medium filled with solid feces Kidneys: slightly enlarged	lungs: low-grade multifocal lymphoplasmacellular pneumonia with a high number of coccoid bacteria within the protein-rich exudate	liver: Achromobacter xylosoxidans (++), Stenotrophomonas maltophilia (+)
			liver, kidney, stomach, small intestine, pancreas: no pathological findings	lung: Achromobacter xylosoxidans (+++), Stenotrophomonas maltophilia (+++)
				kidney: Stenotrophomonas maltophilia (+), *Streptococcus mitis* (+)
				inhibitor substance test: positive
BH171/14/26–29	male	lungs: filled with yellowish exudate, deposits down to the air sacs Stomach: empty Small intestine: slightly filled with greenish mucus Cecum, large intestine: empty	heart, kidney, small intestine, pancreas, brain: no pathological findings	liver: Stenotrophomonas maltophilia (+++)
			lungs: confluent infiltration of lymphocytes and plasma cells, hyperplastic epithelium, protein-rich exudate with a high amount of epithelial and leucocytic cells	lung: Stenotrophomonas maltophilia (+++)
				kidney: Stenotrophomonas maltophilia (+++)
				inhibitor substance test: positive

### Metagenomic sequencing and comparative analysis

**Fig. 1:**
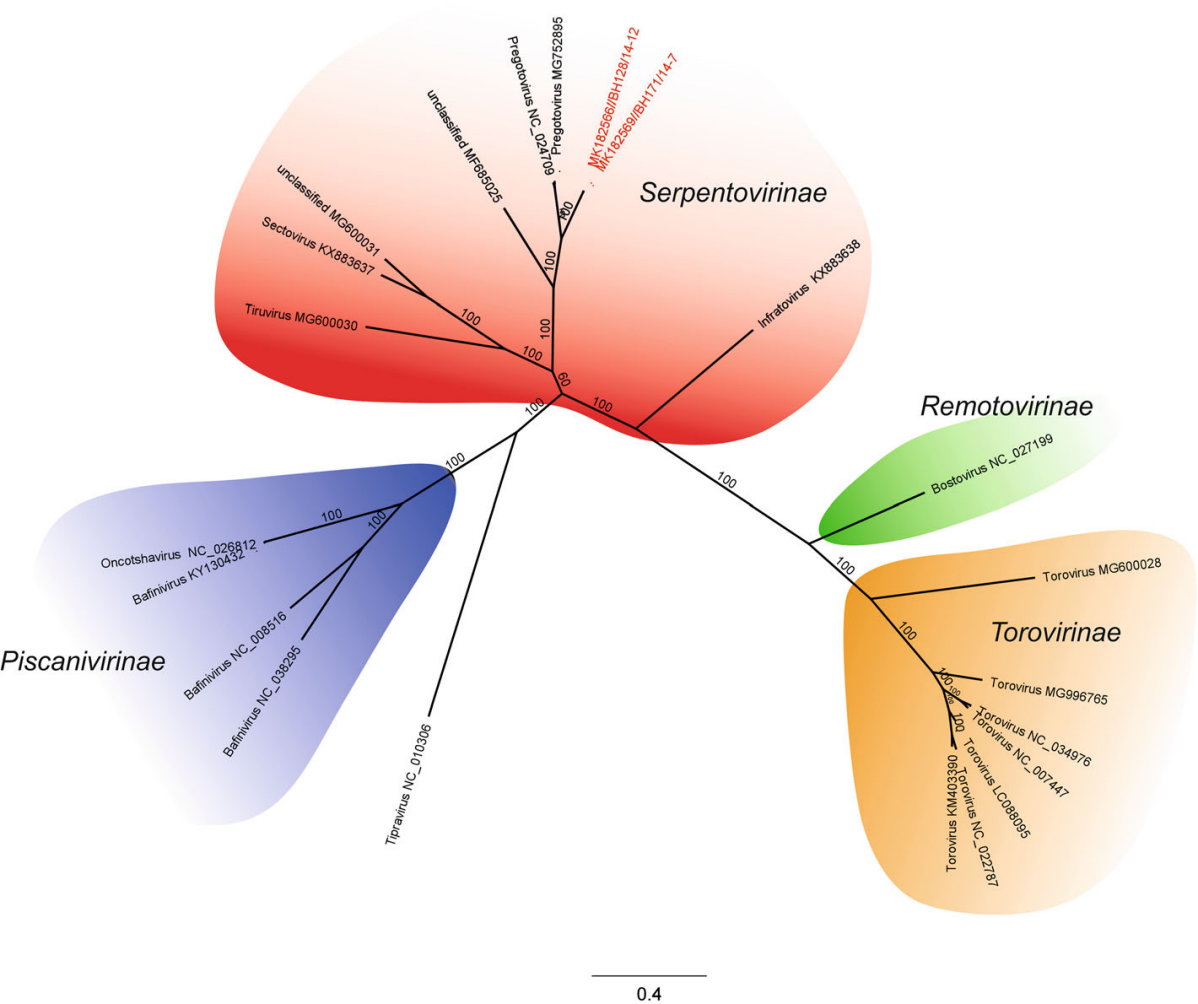
Phylogenetic tree of complete genome nucleotide sequences of the family *Tobaniviridae*, suborder *Tornidoviridae*. The two newly generated snake nidoviruses are highlighted in red

Organ material of one green tree pythons (*Morelia viridis*) was subjected to a metagenome analysis. Thereby, a few reads with the highest overall identity to a ball python snake nidovirus were detected. Besides, reads of a retrovirus could be classified. Afterwards two other snake samples with a higher viral load were chosen for whole genome sequencing. A primer walking approach could close the gaps of the snake nidovirus sequences and thus two complete coding genomes could be generated with a sequence length of 32.88 kb and 32.75 kb respectively. The overall genome organization with eight open reading frames is similar to the other published snake nidoviruses (see Additional file 1: Figure S1). Due to their origin, they were named *Morelia viridis* snake nidovirus. These two sequences share a nucleotide identity of 99.7% to each other. The highest overall sequence similarity to published sequences appears to be 66.8 and 66.9%, respectively to a nidovirus sequence derived from a green tree python in Switzerland (MF351889 [7]). The overall sequence similarities to other published snake nidoviruses is between 64.8 and 65.5%. The ORF1B (part of the replicase gene) seems to be most conserved with sequence similarities on nucleotide level varying between 79.8 and 80.8% and roughly 85% on amino acid level. The most variable area is the ORF3 (glycoprotein) with nucleotide sequence similarities of 53.5–47.5% and amino acid similarities of 40.7–33.2% (Table 3). All reptile nidoviruses cluster together in the genera *Serpentovirinae* inside the family *Tobaniviridae* within the order Nidovirales (Fig. 1).

### Molecular assays and tissue tropism

**Fig. 2:**
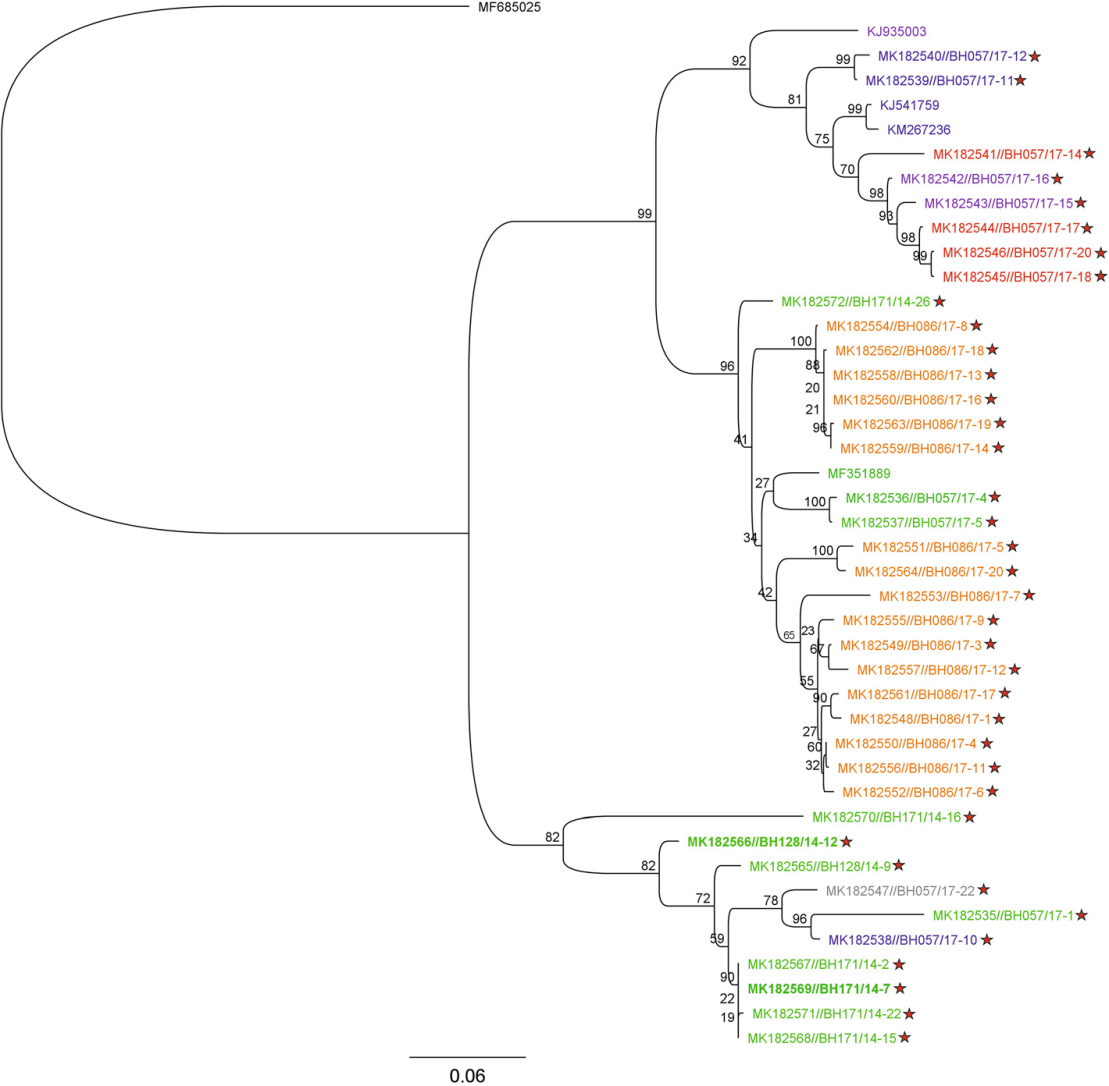
Maximum-likelihood phylogenetic tree of previously published and newly identified reptile nidoviruses within the subfamily *Serpentovirinae*. The tree is based on a 882 bp long nucleotide sequence in the ORF1B coding for the RNA-dependent RNA polymerase. Statistical support for nodes was obtained by bootstrapping (1000 replicates). Accession numbers are indicated after the respective sequence. The sequences are derived from different species: Bellinger River snapping turtle (black), green tree python (green), carpet python (orange), diamond python (red), ball python (blue), indian python (purple) and white-lipped python (grey). Full-length sequences are marked in bold and all newly generated sequences are markes with a red star

To confirm the results from the metagenomic analysis different RT-qPCRs and RT-PCRs for the snake nidoviruses as well as a RT-qPCR for the snake retrovirus were developed. The snake nidovirus RT-qPCRs were compared to other published RT-qPCRs [7] and thereby the results further validated (Additional file 1: Table S1). For this, 60 negative and 46 positive oral swabs were investigated using two different PCRs. Different tissue samples from the nine dissected green tree pythons were tested by RT-qPCR. The highest viral loads could be detected in lung and liver samples matching the histopathologic results whereas intestine and kidney samples were mostly negative or showed discontinuous results (Table 4). To confirm whether the identified snake nidovirus and retrovirus are related to the observed clinical signs, further samples of affected and apparently healthy or otherwise diseased animals were tested (Table 5). The retrovirus could not be found in all affected animals, but instead in animals deceased from a lymphoma (data not shown), which did not exhibit typical symptoms like pneumonia and stomatitis. The snake nidovirus RT-qPCR was positive for most of the animals with pneumonia, but also for a few of asymptomatic snakes (Table 6). To exclude contamination of the RT-qPCR set up an attempt was made to obtain a partial sequence from the RT-qPCR positive samples. The thereby produced sequences (in total 36 sequences, see Additional file 1: Table S2) were not identical, but shared instead a similarity between 99.89 and 79.4% to each other. A phylogenetic analysis showed no specific clustering according to the stock, the animal species or the occurrence of disease in the snake (Fig. 2).

### Screening for snake nidoviruses

Until the end of 2018, a total of 1554 boid snakes were tested by RT-qPCR for snake nidoviruses resulting in 439 positive animals (Table 2). The positive samples originated from all 16 German federal states as well as Denmark, Italy, France and Austria. Only one of the investigated 128 boas showed a positive result, whereas in 438 of 1426 pythons nidoviruses were found. Most of the positive snakes were Green Tree pythons (205 out of 438 positive snakes, 47%), followed by ball pythons and carpet pythons (90 and 91 positive snakes, respectively 21%). For the first time, the presence of nidovirus has been proven in Blood pythons, Ringed pythons, Amesthystine pythons, White lipped pythons, Black headed pythons, Persh pygmy pythons and Boelens pythons (Table 2).

**Table 2:** Nidovirus detection in different snake species

species	common name	total number	positive
*Pythoninae*			
*Morelia viridis*	green tree python	497	205
*Python regius*	ball python	408	90
*Morelia spilota complex*	carpet python, diamond python	372	91
*Python brongersmai*	red blood python	61	27
*Python molurus*	indian python	21	10
*Simalia boeleni*	Boelen’s python	13	6
*Aspidites melanocephalus*	black-headed python	5	3
*Antaresia perthensis*	Perth pygmy python	6	2
*Bothrochilus albertisii*	White lipped python	6	2
*Simalia amethistina*	Amethystine python	3	1
*Bothrochilus boa*	ringed python	3	1
*Others (Malayopython reticulatus, Python anchietae, Malayopython timoriensi, Apodora papuana, Antaresia stimsoni, Antaresia maculosa, Liasis mackloti)*	Reticulated python, Angolan python, Timor python, Papua python, Stimson’s python, Spotted python, Macklot’s python	31	0
		1426	438
*Boidae*			
*Boa constrictor*	common boa	72	1
*Corallus caninus*	emerald tree boa	35	0
*Epicrates cenchria, Corallus hortulanus, Corallus enydris, Corallus batesii, Acrantophis dumerili, Eunectes murinus)*	rainbow boa, garden tree boa, Madagaskar ground boa, green anaconda, Pacific ground boa	21	0
		128	1

Samples were evaluated according to their anamnesis. Anamnesis was classified in typical symptoms or pathology (stomatitis and/or pneumonia), other symptoms or pathology, no symptoms/routine investigations and the group of snakes for which no information was available. The three most common python species providing most samples were evaluated (Green Tree pythons, ball pythons, carpet pythons). From 377 snake nidovirus RNA positive animals 75 showed signs of a disease and 285 did not show any clinical disease (Table 6). In 913 negative animals, 131 snakes revealed clinical symptoms or pathology, whereas 703 were without any symptoms (Table 6). No correlation between symptoms and finding of nidovirus could be established.

Follow-up investigations of a few snakes show a snake nidovirus RNA positive oral swab over several months. In addition, other animals from the same breeding stock never scored positive.

We further validated the new developed real-time PCR by comparing it to the PCR used from Dervas et al. [7]. In total, 60 negative and 46 positive swabs were tested using both methods. Results of our PCR were in line with the PCR developed by Dervas et al. (Additional file 1: Table S1). All positive samples showed comparable results with both PCRs. In two samples, results were negative in the PCR according to Dervas et al., whereas low reactions was found in our PCR assays.

## Discussion

Respiratory diseases are quite a common problem in many collections of boid snakes. Viral agents like paramyxoviruses, arenaviruses and others are able to produce respiratory symptoms. However, in many collections, respiratory disease with high morbidity and mortality was found which was not caused by one of the well-known viruses. In the last years, with the discovery of snake nidoviruses the knowledge about pneumonia in boid snakes improved. These viruses were detected after different pythons succumbed to disease after a few months [4–6]. In our case, the first nidovirus detection occurred in a breeding stock of green tree pythons in which several animals showed severe respiratory signs, purulent stomatitis, poor or non-existing appetite, and weight loss. Mortality rates were high despite supportive treatment and care. Unbiased deep sequencing showed reads of a nidovirus and from two deceased animals full-length sequences could be assembled. These sequences are a little bit shorter than the other published full-length sequences of snakes, but belong still to the longest RNA genomes. The sequence identity to the other published genomes is rather low (< 66.9% on nucleotide sequence) with the highest similarity to the virus described in green tree pythons from Switzerland (Table 3) [7], whereas the three sequences published in 2014 are more similar to each other. Nevertheless, all reptile nidoviruses cluster together within the genus *Pregotovirus* (Fig. 1) [4]. Besides the snake nidovirus, the metagenomics analysis showed reads of a snake retrovirus. This retrovirus could be found in control animals showing no signs of respiratory disease and it is probably an already known endogenous retrovirus without a link to pneumonia [1, 3, 31]. The bacterial findings were not consistent and were probably a matter of secondary infections. No evidence for other pathogens could be found. With a newly developed RT-qPCR different tissues from nine deceased green tree pythons were tested to further investigate the tissue tropism. Thereby, a connection between the degree of histological changes and viral RNA detection was indicated (Tables 1 & 4). The highest viral loads were detected in the lung, whereas the other tested organs showed inconsistent viral RNA amounts. This indicates the respiratory tract as primary location of virus replication, makes the transmission by respiratory secretions possible and further strengthens the usefulness of oral or tracheal swabs as in-vivo sampling method [8]. We used the RT-qPCR for an initial screening for further snake nidovirus infected animals, including some animals deceased from other diseases or even apparently healthy (Table 5). To exclude unspecific amplification and laboratory contaminations, we generated partial sequences of the highly conserved ORF1B. Through this approach, 36 partial nidovirus sequences were obtained. Samples with very low viral loads did not result in a suitable sequence. Sequence comparison showed an identity between 99.89 and 79.4% indicating multiple virus strains. No direct relationship between collections, species or severity of disease is visible (Fig. 2). The host range of these viruses is not known and further virus strains not detectable by the used primer pairs could be possible.

**Table 3:** Nucleotide sequence identity (%) of the two newly generated full-length sequences to other published genomes

	Position: length		Sequence identity	Nucleotide sequence identity			Amino acid sequence identity		
	Morelis viridis	Morelis viridis	between	Python regius	Python morulus	Morelia viridis	Python regius	Python morulus	Morelia viridis
	MK182566	MK182569	MK182566/MK182569	KJ541759	KJ935003	MF351889	KJ541759	KJ935003	MF351889
ORF1A; replicase gene	1.032–17.750: 16.719	1.032–17.666: 16.635	99,47%	58.12/57.95%	59.71/59.51%	61.93/61.72%	54.71/54.56%	54.95/54.85%	57.31/57.81%
ORF1B; replicase gene	17.951–24.667: 6717	17.867–24.583: 6717	100%	80.79%	80.65%	79.81%	85.35%	85.49%	83.92%
ORF2; spike gene	24.776–27.605: 2880	24.642–27.521: 2880	100%	72.33%	71.54%	70.83%	72.25%	72.16%	72.68%
ORF3; glycoprotein gene	27.602–28.372: 771	27.518–28.288: 771	100%	53.54%	49.47%	47.49%	40.77%	38.65%	33.21%
ORF4; matrixprotein gene	28.374–29.030: 657	28.290–28.946: 657	100%	71.84/71.99%	70.62/70.78%	68.33/78.59%	76.15%	74.31%	73.97%
ORF5; nucleoprotein gene	29.044–29.502: 459	28.960–29.418: 459	99,78%	70.13/69.91%	70.99/70.78%	69.48/69.69%	68.63%	68.63%	66.67%
ORF6; glycoprotein gene	29.518–30.483: 966	29.434–30.399: 966	100%	52.88%	54.25%	71.76%	39.47%	41.01%	43.60%
ORF7; glycoprotein gene	30.399–31.901: 1503	30.315–31.817: 1503	100%	66.91%	66.27%	68.12%	61.68%	59.64%	60.24%

**Table 4:** Snake nidovirus RNA distribution in tissues of nine green tree pythons (*Morelia viridis*)

	Green tree python 1	Green tree python 2	Green tree python 3	Green tree python 4	Green tree python 5	Green tree python 6	Green tree python 7	Green tree python 8	Green tree python 9
	BH128/14–1-6	BH128/14–7-10	BH128/14–11-14	BH171/14–1-5	BH171/14–6-10	BH171/14–11-15	BH171/14–16-20	BH171/14–21-25	BH171/14/26–29
lung	++	+	+++	+++	+++	+	(+)	+++	+++
liver	+	+	+++	++	+++	+	+	++	+
intestine	–	–	++	++	++	++	–	++	+
kidney	–	+	+	+	+	–	(+)	++	(+)
brain	+	n.d.	n.d.	++	++	++	++	+++	n.d.

RT-qPCR Cq values < 20 = ++++; 20–25 = +++; 25–30 = ++; 30–3 5 = +; 35–40 = (+); > 40 = −

*n.d.* not done

**Table 5:** Pathologic and molecular findings of selected snakes with different symptoms

Sample number	Species name	Common name	Stock number	Clinical signs	Samples examined	Snake nidovirus RT-qPCR	Snake nidovirus conventional RT-PCR	Accession number
BH128/14–1-6	*Morelia viridis*	green tree python	I	stomatitits and pneumonia	lung, liver, intestine, kidney, brain, pancreas	positive	positive	–
BH128/14–7-10	*Morelia viridis*	green tree python	I	stomatitits and pneumonia	lung, liver, intestine, kidney	positive	positive	MK182565
BH128/14–11-14	*Morelia viridis*	green tree python	I	stomatitits and pneumonia	lung, liver, intestine, kidney	positive	positive	MK182566^a^
BH171/14–1-5	*Morelia viridis*	green tree python	I	stomatitits and pneumonia	lung, liver, intestine, kidney, brain	positive	positive	MK182567
BH171/14–6-10	*Morelia viridis*	green tree python	I	stomatitits and pneumonia	lung, liver, intestine, kidney, brain	positive	positive	MK182569^a^
BH171/14–11-15	*Morelia viridis*	green tree python	I	stomatitits and pneumonia	lung, liver, intestine, kidney, brain	positive	positive	MK182568
BH171/14–16-20	*Morelia viridis*	green tree python	I	stomatitits and pneumonia	lung, liver, intestine, kidney, brain	positive	positive	MK182570
BH171/14–21-25	*Morelia viridis*	green tree python	I	stomatitits and pneumonia	lung, liver, intestine, kidney, brain	positive	positive	MK182571
BH171/14/26–29	*Morelia viridis*	green tree python	I	stomatitits and pneumonia	lung, liver, intestine, kidney	positive	positive	MK182572
BH021/15–1-3	*Python regius*	ball python	II	enteritis, sepsis, cardiac	lung, liver, kidney	negative	negative	–
BH021/15–4-5	*Malayopython reticulatus*	reticulated python	III	sepsis	liver, kidney,	negative	negative	–
BH021/15–6-9	*Atheris squamigera*	green bush viper	IV	sepsis	lung, liver, intestine, kidney	negative	negative	–
BH021/15–10-13	*Morelia spilota variegata*	carpet python	V	lymphoma	lung, liver, intestine, kidney	negative	negative	–
BH057/17–1	*Morelia viridis*	green tree python		stomatitits and pneumonia	lung	positive	positive	MK182535
BH057/17–2	*Morelia viridis*	green tree python		pneumonia	lung	negative	positive	–
BH057/17–3	*Morelia viridis*	green tree python		pneumonia	swab	positive	positive	–
BH057/17–4	*Morelia viridis*	green tree python		pneumonia	swab	positive	positive	MK182536
BH057/17–5	*Morelia viridis*	green tree python		pneumonia	swab	positive	positive	MK182537
BH057/17–6	*Morelia viridis*	green tree python		pneumonia	swab	positive	positive	–
BH057/17–7	*Python regius*	ball python		stomatitits and pneumonia	lung	positive	positive	–
BH057/17–8	*Python regius*	ball python		stomatitits and pneumonia	lung	positive	positive	–
BH057/17–9	*Python regius*	ball python		stomatitits and pneumonia	swab	positive	positive	–
BH057/17–10	*Python regius*	ball python		stomatitits and pneumonia	swab	positive	positive	MK182538
BH057/17–11	*Python regius*	ball python		pneumonia	swab	positive	positive	MK182539
BH057/17–12	*Python regius*	ball python		pneumonia	swab	positive	positive	MK182540
BH057/17–13	*Python regius*	ball python		pneumonia	swab	positive	positive	–
BH057/17–14	*Python molurus*	indian python		stomatitits and pneumonia	lung	positive	positive	MK182541
BH057/17–15	*Python molurus*	indian python		pneumonia	swab	positive	positive	MK182542
BH057/17–16	*Python molurus*	indian python		pneumonia	swab	positive	positive	MK182543
BH057/17–17	*Morelia spilota spilota*	diamond python		stomatitits and pneumonia	lung	positive	positive	MK182544
BH057/17–18	*Morelia spilota spilota*	diamond python		stomatitits and pneumonia	lung	positive	positive	MK182545
BH057/17–19	*Morelia spilota spilota*	diamond python		pneumonia	swab	positive	positive	–
BH057/17–20	*Morelia spilota spilota*	diamond python		pneumonia	swab	positive	positive	MK182546
BH057/17–21	*Bothrochilus albertisii*	white-lipped python		none	swab	positive	positive	–
BH057/17–22	*Bothrochilus albertisii*	white-lipped python		none	swab	positive	positive	MK182547
BH086/17–1	*Morelia spilota*	carpet/diamond python		none	swab	positive	negative	MK182548
BH086/17–2	*Morelia spilota*	carpet/diamond python		none	swab	positive	positive	–
BH086/17–3	*Morelia spilota*	carpet/diamond python		none	swab	positive	negative	MK182549
BH086/17–4	*Morelia spilota*	carpet/diamond python		none	swab	positive	negative	MK182550
BH086/17–5	*Morelia spilota*	carpet/diamond python		none	swab	positive	positive	MK182551
BH086/17–6	*Morelia spilota*	carpet/diamond python		none	swab	positive	negative	MK182552
BH086/17–7	*Morelia spilota*	carpet/diamond python		none	swab	positive	positive	MK182553
BH086/17–8	*Morelia spilota*	carpet/diamond python		none	swab	positive	negative	MK182554
BH086/17–9	*Morelia spilota*	carpet/diamond python		none	swab	positive	positive	MK182555
BH086/17–10	*Morelia spilota*	carpet/diamond python		none	swab	positive	positive	–
BH086/17–11	*Morelia spilota*	carpet/diamond python		none	swab	positive	negative	MK182556
BH086/17–12	*Morelia spilota*	carpet/diamond python		none	swab	positive	negative	MK182557
BH086/17–13	*Morelia spilota*	carpet/diamond python		none	swab	positive	positive	MK182558
BH086/17–14	*Morelia spilota*	carpet/diamond python		none	swab	positive	negative	MK182559
BH086/17–15	*Morelia spilota*	carpet/diamond python		none	swab	negative	negative	–
BH086/17–16	*Morelia spilota*	carpet/diamond python		none	swab	positive	negative	MK182560
BH086/17–17	*Morelia spilota*	carpet/diamond python		none	swab	positive	negative	MK182561
BH086/17–18	*Morelia spilota*	carpet/diamond python		none	swab	positive	negative	MK182562
BH086/17–19	*Morelia spilota*	carpet/diamond python		none	swab	positive	negative	MK182563
BH086/17–20	*Morelia spilota*	carpet/diamond python		none	swab	positive	negative	MK182564

^a^full-length sequence available

Further screening of a total of 1554 animals resulted in 439 nidovirus RNA positive animals (Table 2). From 377 (for which information about the disease status was available) nidovirus RNA positive animals 285 showed no respiratory disease (Table 6). In addition to the species described in previous reports, we could prove the infection in *Python brongersmai*, *Bothrochilus albertisii*, *Brothrochilus boa*, *Morelia boeleni*, *Aspidites melanocephalus* as well as Papua pythons (*Apodora papuana*, data not shown, 2019) further expanding the viral host range. Approximately 31% of all tested pythons were positive. In spite of this, only one boa out of 128 animals revealed the presence of nidovirus genome. This is in concordance to one published study [8]. Unfortunately, no material for sequencing was available from the infected boa. This roughly confirms the 27% positive pythons [8]. At least in our study, the detection of viral RNA correlates not always with clinical signs.

**Table 6:** Symptoms of snake nidovirus RNA positive and negative animals (most frequently examined species)

		Snake nidovirus RNA positive samples					Snake nidovirus RNA negative samples				
Species	Common name	Total positive	Stomatitis and/or respiratory disease	Other disease	No disease (routine investi-gations)	No information	Total negative	Stomatitis and/or respiratory disease	Other disease	No disease (routine investi-gations)	No information
*Morelia viridis*	green tree python	195	39	4	138	14	327	27	20	264	16
*Python regius*	ball python	90	20	1	67	2	309	29	29	240	11
*Morelia spilota complex*	carpet python, diamond python	92	8	3	80	1	277	12	14	249	2
	total	377	67	8	285	17	913	68	63	753	29

Interestingly, in pythons originating from the asian continent, the prevalence of nidovirus was much higher than in other pythons, for example ball pythons (Africa). A total of 41% of the investigated Green Tree pythons were positive for the virus; in Carpet pythons 24% and in Ball pythons 22% were positive, respectively.

Hoon-Hanks et al. fulfilled the Koch’s postulates by experimental infection of ball pythons [10]. Therefore, the detection of nidovirus RNA in apparently healthy individuals may reflect testing during the incubation period or a previous nidovirus infection, because some animals stayed positive in oral swab samples over several months (data not shown). Whether it is infectious virus, or rather a form of RNA persistence is unclear. Other animals from infected collections never turned positive, suggesting a non-airborne transmission. Co-infections or non-pathogenic causes like e.g. stress through newly purchased animals may play a crucial role in the development of clinical disease. No specific treatment is available, infected snakes should be isolated and the testing for nidovirus included in standard diagnostic workup.

## Conclusion

Our results show a nationwide distribution of nidoviruses in Germany with possible many existing strains. In total 439 of 1554 tested snakes were positive for nidovirus but only a few of them revealed clinical signs like stomatitis or severe respiratory disease. Therefore, no obvious correlation between virus and clinical disease could be established. Some of the positive results may be due to testing during the incubation period or samples may have been taken during reconvalescence of a nidovirus infection. Results indicate that a nidovirus infection in pythons may cause no to severe disease possibly depending on the snake species, immune status of the snake, pathogenic potential of the virus strain or other unknown factors. Our investigations show new aspects of a nidovirus infection in pythons and contribute to the understanding of the biology of snake nidoviruses.

## Supplementary information

**Additional file 1: Table S1.** Comparison of the newly developed real-time RT-PCRs with the one published by Dervas et al. (positive samples). **Table S2.** Sequence identity between all in this study generated partial sequences. **Figure S1.** Genome organization of snake nidoviruses. Sequences marked in red were generated within this study.

## Data Availability

All relevant information is provided in this current manuscript.

## Abbreviations

ICTV: International Committee on Taxonomy of Viruses
MAFFT: Multiple alignment using fast Fourier transform
MALDI TOF: Matrix Assisted Laser Desorption Ionization - Time of Flight
PPRSV: Porcine reproductive and respiratory syndrome virus
SARS: Severe acute respiratory syndrome
