# Supplementary material for: Investigations into the presence of nidoviruses in pythons
Source: Virol J. 2020 Jan 17;17:6. doi: 10.1186/s12985-020-1279-5 (PMC6969405; doi:10.1186/s12985-020-1279-5)

## Additional file 1

Table S1: Comparison of the newly developed real-time RT-PCRs with the one published by Dervas et al. (positive samples)

| sample number | Species | This study | Dervas et al. 2017 |
| --- | --- | --- | --- |
| 118171 | Ball python | 26.86 | 28.81 |
| 118208 | Burmese python | 33.33 | 29.69 |
| 118696 | Ball python | 29.17 | 25.39 |
| 118697 | Ball python | 28.52 | 26.49 |
| 119395 | Green Tree python | 23.36 | 23.50 |
| 121321 | Ball python | 30.67 | 30.5 |
| 121325 | Ball python | 29.05 | 31.99 |
| 121326 | Burmese python | 27.92 | 26.30 |
| 122336 | Green Tree python | 17.37 | 17.65 |
| 123279 | Green Tree python | 22.94 | 22.22 |
| 123282 | Green Tree python | 25.15 | 25.1 |
| 123284 | Green Tree python | 23.69 | 23.72 |
| 123285 | Green Tree python | 22.12 | 22.27 |
| 124075 | Ball python | 21.24 | 23.25 |
| 124076 | Ball python | 25.84 | 27.23 |
| 128183 | Green Tree python | 21.37 | 20.19 |
| 128184 | Green Tree python | 20.51 | 20.93 |
| 128185 | Green Tree python | 23.40 | 24.14 |
| 131981 | Carpet python | 15.81 | 13.85 |
| 132578 | Ball python | 25.81 | 25.45 |
| 132579 | Ball python | 28.95 | 30.00 |
| 133859 | Green Tree python | 27.93 | 26.39 |
| 133860 | Green Tree python | 24.23 | 21.4 |
| 137237 | Green Tree python | 26.54 | 29.83 |
| 137239 | Green Tree python | 21.94 | 23.85 |
| 137240 | Green Tree python | 26.38 | 27.35 |
| 137241 | Green Tree python | 16.68 | 14.18 |
| 137242 | Green Tree python | 20.52 | 17.95 |
| 138139 | Papua python | 35.33 | 34.24 |
| 138140 | Green Tree python | 22.63 | 21.01 |
| 139276 | Blood python | 19.82 | 18.97 |
| 139291 | Green Tree python | 16.99 | 19.37 |
| 139292 | Green Tree python | 18.94 | 19.25 |
| 139895 | Burmese python | 31.8 | 31.3 |
| 141487 | Carpet python | 19.6 | 21.3 |
| 141850 | Green Tree python | 27.2 | 26.7 |
| 143728 | Green Tree python | 20.9 | 19.9 |
| 143928 | Green Tree python | 22.2 | 22.1 |
| 144212 | Carpet python | 32.5 | 31.1 |
| 144220 | Carpet python | 23.7 | 21.4 |
| 144820 | Green Tree python | 28.4 | 30.7 |
| 145075 | Ball python | 28.7 | 30.5 |
| 145487 | Green Tree python | 31.2 | 27.6 |
| 148673 | Green Tree python | 24.2 | 24.2 |

Table S2: Sequence identity between all in this study generated partial sequences.

|  |  | MK182535//BH057/17-1 | MK182538//BH057/17-10 | MK182547//BH057/17-22 | MK182567//BH171/14-2 | MK182568//BH171/14-15 | MK182569//BH171/14-7 | MK182571//BH171/14-22 | MK182565//BH128/14-9 | MK182566//BH128/14-12 | MK182570//BH171/14-16 | MK182536//BH057/17-4 | MK182537//BH057/17-5 | MK182548//BH086/17-1 | MK182561//BH086/17-17 | MK182549//BH086/17-3 | MK182550//BH086/17-4 | MK182556//BH086/17-11 | MK182552//BH086/17-6 | MK182555//BH086/17-9 | MK182557//BH086/17-12 | MK182572//BH171/14-26 | MK182553//BH086/17-7 | MK182554//BH086/17-8 | MK182558//BH086/17-13 | MK182560//BH086/17-16 | MK182562//BH086/17-18 | MK182559//BH086/17-14 | MK182563//BH086/17-19 | MK182551//BH086/17-5 | MK182564//BH086/17-20 | MK182539//BH057/17-11 | MK182540//BH057/17-12 | MK182541//BH057/17-14 | MK182543//BH057/17-15 | MK182542//BH057/17-16 | MK182544//BH057/17-17 | MK182545//BH057/17-18 | MK182546//BH057/17-20 |
| --- | --- | --- | --- | --- | --- | --- | --- | --- | --- | --- | --- | --- | --- | --- | --- | --- | --- | --- | --- | --- | --- | --- | --- | --- | --- | --- | --- | --- | --- | --- | --- | --- | --- | --- | --- | --- | --- | --- | --- |
|  |  | Morelia viridis | Morelia viridis | Bothrochilus albertisii | Morelia viridis | Morelia viridis | Morelia viridis | Morelia viridis | Morelia viridis | Morelia viridis | Morelia viridis | Morelia viridis | Morelia viridis | Morelia viridis | Morelia spilota | Morelia spilota | Morelia spilota | Morelia spilota | Morelia spilota | Morelia spilota | Morelia spilota | Morelia viridis | Morelia spilota | Morelia spilota | Morelia spilota | Morelia spilota | Morelia spilota | Morelia spilota | Morelia spilota | Morelia spilota | Morelia spilota | Morelia viridis | Morelia viridis | Python molurus | Python molurus | Python molurus | Morelia spilota | Morelia spilota | Morelia spilota |
| MK182535//BH057/17-1 | Morelia viridis |  | 95.0 | 93.2 | 92.0 | 92.0 | 92.0 | 91.9 | 91.1 | 90.0 | 86.2 | 80.2 | 80.1 | 80.2 | 80.2 | 80.7 | 80.5 | 80.4 | 80.5 | 80.7 | 80.4 | 80.5 | 79.3 | 80.7 | 80.9 | 80.9 | 81.0 | 80.8 | 80.7 | 80.2 | 80.4 | 80.0 | 79.9 | 80.2 | 79.9 | 80.3 | 79.6 | 79.4 | 79.4 |
| MK182538//BH057/17-10 | Morelia viridis | 95.0 |  | 96.9 | 95.7 | 95.7 | 95.7 | 95.6 | 94.8 | 93.7 | 88.2 | 82.5 | 82.5 | 83.0 | 83.0 | 83.4 | 83.3 | 83.1 | 83.0 | 83.3 | 83.0 | 83.4 | 81.7 | 83.5 | 83.4 | 83.4 | 83.3 | 83.3 | 83.1 | 82.5 | 82.7 | 82.8 | 82.8 | 83.3 | 82.7 | 83.1 | 82.7 | 82.7 | 82.7 |
| MK182547//BH057/17-22 | Bothrochilus albertisii | 93.2 | 96.9 |  | 95.9 | 95.9 | 95.9 | 95.9 | 94.7 | 93.3 | 88.3 | 81.4 | 81.4 | 82.2 | 82.2 | 82.6 | 82.5 | 82.4 | 82.2 | 82.5 | 82.2 | 82.8 | 80.5 | 82.7 | 82.8 | 82.8 | 82.7 | 82.7 | 82.6 | 81.8 | 82.0 | 82.2 | 82.2 | 82.9 | 82.4 | 82.8 | 82.1 | 82.1 | 82.1 |
| MK182567//BH171/14-2 | Morelia viridis | 92.0 | 95.7 | 95.9 |  | 100.0 | 100.0 | 99.8 | 97.7 | 96.2 | 87.8 | 82.6 | 82.6 | 82.4 | 82.6 | 82.7 | 82.6 | 82.5 | 82.4 | 82.8 | 82.4 | 82.8 | 80.5 | 82.9 | 82.8 | 82.8 | 82.7 | 82.7 | 82.6 | 81.7 | 81.7 | 82.0 | 82.0 | 82.6 | 82.8 | 83.1 | 82.6 | 82.1 | 82.1 |
| MK182568//BH171/14-15 | Morelia viridis | 92.0 | 95.7 | 95.9 | 100.0 |  | 100.0 | 99.8 | 97.7 | 96.2 | 87.8 | 82.6 | 82.6 | 82.4 | 82.6 | 82.7 | 82.6 | 82.5 | 82.4 | 82.8 | 82.4 | 82.8 | 80.5 | 82.9 | 82.8 | 82.8 | 82.7 | 82.7 | 82.6 | 81.7 | 81.7 | 82.0 | 82.0 | 82.6 | 82.8 | 83.1 | 82.6 | 82.1 | 82.1 |
| MK182569//BH171/14-7 | Morelia viridis | 92.0 | 95.7 | 95.9 | 100.0 | 100.0 |  | 99.8 | 97.7 | 96.2 | 87.8 | 82.6 | 82.6 | 82.4 | 82.6 | 82.7 | 82.6 | 82.5 | 82.4 | 82.8 | 82.4 | 82.8 | 80.5 | 82.9 | 82.8 | 82.8 | 82.7 | 82.7 | 82.6 | 81.7 | 81.7 | 82.0 | 82.0 | 82.6 | 82.8 | 83.1 | 82.6 | 82.1 | 82.1 |
| MK182571//BH171/14-22 | Morelia viridis | 91.9 | 95.6 | 95.9 | 99.8 | 99.8 | 99.8 |  | 97.6 | 96.0 | 87.6 | 82.4 | 82.4 | 82.1 | 82.4 | 82.5 | 82.4 | 82.2 | 82.1 | 82.6 | 82.1 | 82.6 | 80.3 | 82.7 | 82.6 | 82.6 | 82.5 | 82.5 | 82.4 | 81.4 | 81.4 | 81.9 | 81.9 | 82.4 | 82.6 | 82.9 | 82.4 | 81.9 | 81.9 |
| MK182565//BH128/14-9 | Morelia viridis | 91.1 | 94.8 | 94.7 | 97.7 | 97.7 | 97.7 | 97.6 |  | 95.7 | 87.1 | 83.7 | 83.7 | 83.8 | 84.0 | 83.9 | 84.2 | 84.0 | 83.9 | 83.7 | 83.7 | 84.3 | 81.9 | 84.5 | 84.4 | 84.4 | 84.3 | 84.3 | 84.2 | 82.8 | 83.0 | 82.5 | 82.5 | 82.6 | 83.0 | 83.4 | 82.8 | 82.4 | 82.4 |
| MK182566//BH128/14-12 | Morelia viridis | 90.0 | 93.7 | 93.3 | 96.2 | 96.2 | 96.2 | 96.0 | 95.7 |  | 87.9 | 85.0 | 85.0 | 85.4 | 85.6 | 85.6 | 85.6 | 85.5 | 85.4 | 85.6 | 85.4 | 85.7 | 83.5 | 85.9 | 85.7 | 85.7 | 85.6 | 85.5 | 85.4 | 84.2 | 84.2 | 83.6 | 83.6 | 83.6 | 83.9 | 84.3 | 83.9 | 83.5 | 83.5 |
| MK182570//BH171/14-16 | Morelia viridis | 86.2 | 88.2 | 88.3 | 87.8 | 87.8 | 87.8 | 87.6 | 87.1 | 87.9 |  | 82.4 | 82.4 | 82.0 | 82.0 | 82.4 | 82.4 | 82.2 | 82.6 | 83.0 | 82.8 | 82.8 | 80.8 | 82.8 | 83.0 | 83.0 | 83.1 | 82.8 | 82.7 | 82.0 | 82.2 | 81.4 | 81.2 | 82.1 | 81.6 | 82.7 | 81.9 | 81.2 | 81.2 |
| MK182536//BH057/17-4 | Morelia viridis | 80.2 | 82.5 | 81.4 | 82.6 | 82.6 | 82.6 | 82.4 | 83.7 | 85.0 | 82.4 |  | 99.5 | 95.1 | 94.8 | 94.8 | 95.1 | 95.0 | 95.5 | 94.9 | 94.7 | 95.1 | 93.0 | 94.7 | 94.5 | 94.5 | 94.6 | 94.2 | 94.1 | 93.7 | 94.1 | 88.8 | 88.3 | 87.4 | 87.0 | 86.9 | 86.2 | 86.0 | 86.0 |
| MK182537//BH057/17-5 | Morelia viridis | 80.1 | 82.5 | 81.4 | 82.6 | 82.6 | 82.6 | 82.4 | 83.7 | 85.0 | 82.4 | 99.5 |  | 95.4 | 95.0 | 95.0 | 95.4 | 95.2 | 95.7 | 95.1 | 94.9 | 95.4 | 93.2 | 94.7 | 94.5 | 94.5 | 94.6 | 94.2 | 94.1 | 93.9 | 94.3 | 89.0 | 88.6 | 87.4 | 87.0 | 86.9 | 86.2 | 86.0 | 86.0 |
| MK182548//BH086/17-1 | Morelia viridis | 80.2 | 83.0 | 82.2 | 82.4 | 82.4 | 82.4 | 82.1 | 83.8 | 85.4 | 82.0 | 95.1 | 95.4 |  | 99.2 | 98.5 | 98.9 | 98.8 | 98.8 | 98.2 | 98.0 | 95.7 | 95.4 | 94.5 | 94.0 | 94.0 | 93.9 | 94.0 | 93.9 | 94.5 | 94.5 | 88.9 | 88.5 | 87.7 | 87.0 | 87.1 | 86.7 | 86.7 | 86.4 |
| MK182561//BH086/17-17 | Morelia spilota | 80.2 | 83.0 | 82.2 | 82.6 | 82.6 | 82.6 | 82.4 | 84.0 | 85.6 | 82.0 | 94.8 | 95.0 | 99.2 |  | 98.6 | 99.0 | 98.9 | 98.9 | 98.3 | 97.9 | 95.6 | 95.7 | 94.6 | 94.1 | 94.1 | 94.0 | 94.1 | 94.0 | 94.3 | 94.3 | 88.8 | 88.3 | 87.1 | 86.7 | 87.0 | 86.5 | 86.5 | 86.3 |
| MK182549//BH086/17-3 | Morelia spilota | 80.7 | 83.4 | 82.6 | 82.7 | 82.7 | 82.7 | 82.5 | 83.9 | 85.6 | 82.4 | 94.8 | 95.0 | 98.5 | 98.6 |  | 99.0 | 98.9 | 98.6 | 98.8 | 99.0 | 95.8 | 95.7 | 94.8 | 94.3 | 94.3 | 94.2 | 94.3 | 94.2 | 95.0 | 95.0 | 89.3 | 88.8 | 87.9 | 87.3 | 87.4 | 87.2 | 87.2 | 87.0 |
| MK182550//BH086/17-4 | Morelia spilota | 80.5 | 83.3 | 82.5 | 82.6 | 82.6 | 82.6 | 82.4 | 84.2 | 85.6 | 82.4 | 95.1 | 95.4 | 98.9 | 99.0 | 99.0 |  | 99.9 | 99.4 | 99.1 | 98.2 | 96.5 | 95.8 | 95.1 | 94.7 | 94.7 | 94.6 | 94.7 | 94.6 | 94.9 | 94.9 | 89.3 | 88.8 | 87.8 | 87.4 | 87.6 | 87.1 | 87.1 | 86.9 |
| MK182556//BH086/17-11 | Morelia spilota | 80.4 | 83.1 | 82.4 | 82.5 | 82.5 | 82.5 | 82.2 | 84.0 | 85.5 | 82.2 | 95.0 | 95.2 | 98.8 | 98.9 | 98.9 | 99.9 |  | 99.3 | 99.0 | 98.1 | 96.4 | 95.7 | 95.0 | 94.6 | 94.6 | 94.5 | 94.6 | 94.5 | 94.8 | 94.8 | 89.1 | 88.7 | 87.7 | 87.3 | 87.4 | 87.0 | 87.0 | 86.8 |
| MK182552//BH086/17-6 | Morelia spilota | 80.5 | 83.0 | 82.2 | 82.4 | 82.4 | 82.4 | 82.1 | 83.9 | 85.4 | 82.6 | 95.5 | 95.7 | 98.8 | 98.9 | 98.6 | 99.4 | 99.3 |  | 98.5 | 98.5 | 96.2 | 96.2 | 95.0 | 94.8 | 94.8 | 94.7 | 94.8 | 94.7 | 94.8 | 94.8 | 88.8 | 88.3 | 87.8 | 87.2 | 87.6 | 87.1 | 87.1 | 86.9 |
| MK182555//BH086/17-9 | Morelia spilota | 80.7 | 83.3 | 82.5 | 82.8 | 82.8 | 82.8 | 82.6 | 83.7 | 85.6 | 83.0 | 94.9 | 95.1 | 98.2 | 98.3 | 98.8 | 99.1 | 99.0 | 98.5 |  | 98.4 | 95.9 | 95.6 | 94.7 | 94.2 | 94.2 | 94.3 | 94.2 | 94.1 | 95.1 | 94.9 | 89.7 | 89.3 | 88.3 | 88.0 | 88.1 | 87.4 | 87.4 | 87.2 |
| MK182557//BH086/17-12 | Morelia spilota | 80.4 | 83.0 | 82.2 | 82.4 | 82.4 | 82.4 | 82.1 | 83.7 | 85.4 | 82.8 | 94.7 | 94.9 | 98.0 | 97.9 | 99.0 | 98.2 | 98.1 | 98.5 | 98.4 |  | 95.5 | 95.4 | 94.7 | 94.5 | 94.5 | 94.3 | 94.5 | 94.3 | 94.5 | 94.5 | 88.7 | 88.2 | 87.8 | 87.3 | 87.4 | 87.2 | 87.2 | 87.0 |
| MK182572//BH171/14-26 | Morelia viridis | 80.5 | 83.4 | 82.8 | 82.8 | 82.8 | 82.8 | 82.6 | 84.3 | 85.7 | 82.8 | 95.1 | 95.4 | 95.7 | 95.6 | 95.8 | 96.5 | 96.4 | 96.2 | 95.9 | 95.5 |  | 94.1 | 95.1 | 94.9 | 94.9 | 94.8 | 94.7 | 94.6 | 94.2 | 94.7 | 90.6 | 90.2 | 88.7 | 88.2 | 88.1 | 87.4 | 87.4 | 87.2 |
| MK182553//BH086/17-7 | Morelia spilota | 79.3 | 81.7 | 80.5 | 80.5 | 80.5 | 80.5 | 80.3 | 81.9 | 83.5 | 80.8 | 93.0 | 93.2 | 95.4 | 95.7 | 95.7 | 95.8 | 95.7 | 96.2 | 95.6 | 95.4 | 94.1 |  | 93.4 | 93.2 | 93.2 | 93.1 | 93.2 | 93.1 | 94.5 | 94.0 | 88.2 | 87.7 | 86.4 | 86.0 | 86.3 | 85.9 | 85.6 | 85.6 |
| MK182554//BH086/17-8 | Morelia spilota | 80.7 | 83.5 | 82.7 | 82.9 | 82.9 | 82.9 | 82.7 | 84.5 | 85.9 | 82.8 | 94.7 | 94.7 | 94.5 | 94.6 | 94.8 | 95.1 | 95.0 | 95.0 | 94.7 | 94.7 | 95.1 | 93.4 |  | 99.5 | 99.5 | 99.4 | 99.3 | 99.2 | 93.7 | 94.6 | 88.7 | 88.2 | 87.4 | 87.1 | 87.0 | 86.8 | 86.3 | 86.3 |
| MK182558//BH086/17-13 | Morelia spilota | 80.9 | 83.4 | 82.8 | 82.8 | 82.8 | 82.8 | 82.6 | 84.4 | 85.7 | 83.0 | 94.5 | 94.5 | 94.0 | 94.1 | 94.3 | 94.7 | 94.6 | 94.8 | 94.2 | 94.5 | 94.9 | 93.2 | 99.5 |  | 100.0 | 99.9 | 99.7 | 99.5 | 93.8 | 94.6 | 88.6 | 88.1 | 87.3 | 87.0 | 86.9 | 86.7 | 86.2 | 86.2 |
| MK182560//BH086/17-16 | Morelia spilota | 80.9 | 83.4 | 82.8 | 82.8 | 82.8 | 82.8 | 82.6 | 84.4 | 85.7 | 83.0 | 94.5 | 94.5 | 94.0 | 94.1 | 94.3 | 94.7 | 94.6 | 94.8 | 94.2 | 94.5 | 94.9 | 93.2 | 99.5 | 100.0 |  | 99.9 | 99.7 | 99.5 | 93.8 | 94.6 | 88.6 | 88.1 | 87.3 | 87.0 | 86.9 | 86.7 | 86.2 | 86.2 |
| MK182562//BH086/17-18 | Morelia spilota | 81.0 | 83.3 | 82.7 | 82.7 | 82.7 | 82.7 | 82.5 | 84.3 | 85.6 | 83.1 | 94.6 | 94.6 | 93.9 | 94.0 | 94.2 | 94.6 | 94.5 | 94.7 | 94.3 | 94.3 | 94.8 | 93.1 | 99.4 | 99.9 | 99.9 |  | 99.5 | 99.4 | 93.9 | 94.7 | 88.7 | 88.2 | 87.4 | 87.1 | 87.0 | 86.5 | 86.1 | 86.1 |
| MK182559//BH086/17-14 | Morelia spilota | 80.8 | 83.3 | 82.7 | 82.7 | 82.7 | 82.7 | 82.5 | 84.3 | 85.5 | 82.8 | 94.2 | 94.2 | 94.0 | 94.1 | 94.3 | 94.7 | 94.6 | 94.8 | 94.2 | 94.5 | 94.7 | 93.2 | 99.3 | 99.7 | 99.7 | 99.5 |  | 99.9 | 93.7 | 94.3 | 88.3 | 87.9 | 87.1 | 86.8 | 86.7 | 86.4 | 86.0 | 86.0 |
| MK182563//BH086/17-19 | Morelia spilota | 80.7 | 83.1 | 82.6 | 82.6 | 82.6 | 82.6 | 82.4 | 84.2 | 85.4 | 82.7 | 94.1 | 94.1 | 93.9 | 94.0 | 94.2 | 94.6 | 94.5 | 94.7 | 94.1 | 94.3 | 94.6 | 93.1 | 99.2 | 99.5 | 99.5 | 99.4 | 99.9 |  | 93.8 | 94.5 | 88.2 | 87.8 | 87.0 | 86.7 | 86.5 | 86.3 | 85.9 | 85.9 |
| MK182551//BH086/17-5 | Morelia spilota | 80.2 | 82.5 | 81.8 | 81.7 | 81.7 | 81.7 | 81.4 | 82.8 | 84.2 | 82.0 | 93.7 | 93.9 | 94.5 | 94.3 | 95.0 | 94.9 | 94.8 | 94.8 | 95.1 | 94.5 | 94.2 | 94.5 | 93.7 | 93.8 | 93.8 | 93.9 | 93.7 | 93.8 |  | 98.9 | 88.8 | 88.3 | 86.5 | 86.5 | 86.4 | 86.0 | 85.7 | 86.0 |
| MK182564//BH086/17-20 | Morelia spilota | 80.4 | 82.7 | 82.0 | 81.7 | 81.7 | 81.7 | 81.4 | 83.0 | 84.2 | 82.2 | 94.1 | 94.3 | 94.5 | 94.3 | 95.0 | 94.9 | 94.8 | 94.8 | 94.9 | 94.5 | 94.7 | 94.0 | 94.6 | 94.6 | 94.6 | 94.7 | 94.3 | 94.5 | 98.9 |  | 88.6 | 88.1 | 86.3 | 86.3 | 86.2 | 85.7 | 85.5 | 85.7 |
| MK182539//BH057/17-11 | Morelia viridis | 80.0 | 82.8 | 82.2 | 82.0 | 82.0 | 82.0 | 81.9 | 82.5 | 83.6 | 81.4 | 88.8 | 89.0 | 88.9 | 88.8 | 89.3 | 89.3 | 89.1 | 88.8 | 89.7 | 88.7 | 90.6 | 88.2 | 88.7 | 88.6 | 88.6 | 88.7 | 88.3 | 88.2 | 88.8 | 88.6 |  | 99.2 | 94.0 | 93.8 | 94.1 | 93.0 | 92.9 | 92.9 |
| MK182540//BH057/17-12 | Morelia viridis | 79.9 | 82.8 | 82.2 | 82.0 | 82.0 | 82.0 | 81.9 | 82.5 | 83.6 | 81.2 | 88.3 | 88.6 | 88.5 | 88.3 | 88.8 | 88.8 | 88.7 | 88.3 | 89.3 | 88.2 | 90.2 | 87.7 | 88.2 | 88.1 | 88.1 | 88.2 | 87.9 | 87.8 | 88.3 | 88.1 | 99.2 |  | 93.9 | 93.4 | 93.8 | 92.6 | 92.5 | 92.5 |
| MK182541//BH057/17-14 | Python molurus | 80.2 | 83.3 | 82.9 | 82.6 | 82.6 | 82.6 | 82.4 | 82.6 | 83.6 | 82.1 | 87.4 | 87.4 | 87.7 | 87.1 | 87.9 | 87.8 | 87.7 | 87.8 | 88.3 | 87.8 | 88.7 | 86.4 | 87.4 | 87.3 | 87.3 | 87.4 | 87.1 | 87.0 | 86.5 | 86.3 | 94.0 | 93.9 |  | 94.8 | 95.8 | 94.6 | 94.5 | 94.5 |
| MK182543//BH057/17-15 | Python molurus | 79.9 | 82.7 | 82.4 | 82.8 | 82.8 | 82.8 | 82.6 | 83.0 | 83.9 | 81.6 | 87.0 | 87.0 | 87.0 | 86.7 | 87.3 | 87.4 | 87.3 | 87.2 | 88.0 | 87.3 | 88.2 | 86.0 | 87.1 | 87.0 | 87.0 | 87.1 | 86.8 | 86.7 | 86.5 | 86.3 | 93.8 | 93.4 | 94.8 |  | 98.5 | 98.0 | 97.9 | 97.9 |
| MK182542//BH057/17-16 | Python molurus | 80.3 | 83.1 | 82.8 | 83.1 | 83.1 | 83.1 | 82.9 | 83.4 | 84.3 | 82.7 | 86.9 | 86.9 | 87.1 | 87.0 | 87.4 | 87.6 | 87.4 | 87.6 | 88.1 | 87.4 | 88.1 | 86.3 | 87.0 | 86.9 | 86.9 | 87.0 | 86.7 | 86.5 | 86.4 | 86.2 | 94.1 | 93.8 | 95.8 | 98.5 |  | 98.3 | 97.7 | 97.7 |
| MK182544//BH057/17-17 | Morelia spilota | 79.6 | 82.7 | 82.1 | 82.6 | 82.6 | 82.6 | 82.4 | 82.8 | 83.9 | 81.9 | 86.2 | 86.2 | 86.7 | 86.5 | 87.2 | 87.1 | 87.0 | 87.1 | 87.4 | 87.2 | 87.4 | 85.9 | 86.8 | 86.7 | 86.7 | 86.5 | 86.4 | 86.3 | 86.0 | 85.7 | 93.0 | 92.6 | 94.6 | 98.0 | 98.3 |  | 99.2 | 99.2 |
| MK182545//BH057/17-18 | Morelia spilota | 79.4 | 82.7 | 82.1 | 82.1 | 82.1 | 82.1 | 81.9 | 82.4 | 83.5 | 81.2 | 86.0 | 86.0 | 86.7 | 86.5 | 87.2 | 87.1 | 87.0 | 87.1 | 87.4 | 87.2 | 87.4 | 85.6 | 86.3 | 86.2 | 86.2 | 86.1 | 86.0 | 85.9 | 85.7 | 85.5 | 92.9 | 92.5 | 94.5 | 97.9 | 97.7 | 99.2 |  | 99.8 |
| MK182546//BH057/17-20 | Morelia spilota | 79.4 | 82.7 | 82.1 | 82.1 | 82.1 | 82.1 | 81.9 | 82.4 | 83.5 | 81.2 | 86.0 | 86.0 | 86.4 | 86.3 | 87.0 | 86.9 | 86.8 | 86.9 | 87.2 | 87.0 | 87.2 | 85.6 | 86.3 | 86.2 | 86.2 | 86.1 | 86.0 | 85.9 | 86.0 | 85.7 | 92.9 | 92.5 | 94.5 | 97.9 | 97.7 | 99.2 | 99.8 |  |

Figure S1: Genome organization of snake nidoviruses. Sequences marked in red were generated within this study.


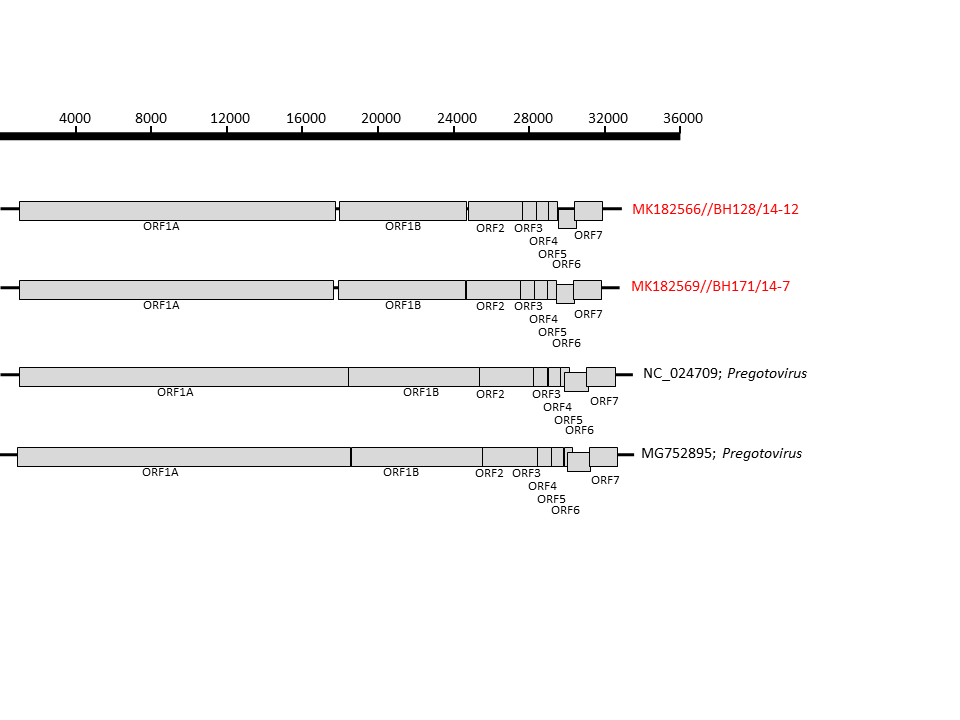

Supplement: Supplementary file 1 — Additional file 1: Table S1. Comparison of the newly developed real-time RT-PCRs with the one published by Dervas et al. (positive samples). Table S2. Sequence identity between all in this study generated partial sequences. Figure S1. Genome organization of snake nidoviruses. Sequences marked in red were generated within this study. [file 12985_2020_1279_MOESM1_ESM.docx]
